# Characterization of age-associated gene expression changes in mouse sweat glands

**DOI:** 10.18632/aging.205776

**Published:** 2024-04-17

**Authors:** Alexandra G. Zonnefeld, Chang-Yi Cui, Dimitrios Tsitsipatis, Yulan Piao, Jinshui Fan, Krystyna Mazan-Mamczarz, Yutong Xue, Fred E. Indig, Supriyo De, Myriam Gorospe

**Affiliations:** 1 Laboratory of Genetics and Genomics, National Institute on Aging Intramural Research Program, National Institutes of Health, Baltimore, MD 21224, USA; 2 Confocal Imaging Core Facility, National Institute on Aging Intramural Research Program, National Institutes of Health, Baltimore, MD 21224, USA

**Keywords:** FOXA1, BEST2, FOXC1, ectodysplasin/Eda, Tabby

## Abstract

Evaporation of sweat on the skin surface is the major mechanism for dissipating heat in humans. The secretory capacity of sweat glands (SWGs) declines during aging, leading to heat intolerance in the elderly, but the mechanisms responsible for this decline are poorly understood. We investigated the molecular changes accompanying SWG aging in mice, where sweat tests confirmed a significant reduction of active SWGs in old mice relative to young mice. We first identified SWG-enriched mRNAs by comparing the skin transcriptome of *Eda* mutant Tabby male mice, which lack SWGs, with that of wild-type control mice by RNA-sequencing analysis. This comparison revealed 171 mRNAs enriched in SWGs, including 47 mRNAs encoding ‘core secretory’ proteins such as transcription factors, ion channels, ion transporters, and trans-synaptic signaling proteins. Among these, 28 SWG-enriched mRNAs showed significantly altered abundance in the aged male footpad skin, and 11 of them, including *Foxa1, Best2, Chrm3*, and *Foxc1* mRNAs, were found in the ‘core secretory’ category. Consistent with the changes in mRNA expression levels, immunohistology revealed that higher numbers of secretory cells from old SWGs express the transcription factor FOXC1, the protein product of *Foxc1* mRNA. In sum, our study identified mRNAs enriched in SWGs, including those that encode core secretory proteins, and altered abundance of these mRNAs and proteins with aging in mouse SWGs.

## INTRODUCTION

In humans, sweat glands (SWGs) play a pivotal role in maintaining body temperature within a narrow window. Each SWG is a tiny single tubular structure, comprised of a secretory coil in the deep dermis and a reabsorption duct connecting the secretory coil to the skin surface [1]. Millions of SWGs are distributed across the human skin and can secrete liters of sweat per day [2]. The primary sweat produced in the secretory coil is isotonic with blood plasma, but the final sweat secreted to the skin surface is hypotonic due to partial reabsorption of ions in the duct region [3]. The final sweat is primarily water, and its evaporation from the skin surface effectively cools the body. Sweat secretion is carefully orchestrated by the coordinated action of transcription factors, ion channels, ion cotransporters, and neurotransmitters; the FOXA1 transcription factor was recently shown to be a critical regulator of the secretory process [4–6].

With advancing age, the secretory capacity of SWGs declines, leading to heat intolerance in the elderly [7, 8]. In this regard, the progressive increase of the elderly populations, together with global warming trends, have led to an escalation in incidence of heat-related illnesses [9]. Between 2018 and 2020, a total of 3066 heat-related deaths were reported in the USA alone (https://www.cdc.gov/mmwr/), and older people are much more vulnerable, as indicated by a recent analysis across a broad population in Europe [10]. Therefore, understanding the mechanisms of age-related deterioration in SWGs is crucial in order to reduce health issues caused by a hot environment.

The morphology of SWGs changes during aging, twisting, rotating, and moving up toward the skin surface due to shrinking dermal thickness [11], consistent with the functional decline of sweat secretion with age [7, 8]. However, the molecular mechanisms underlying the decline in secretory function of aging SWGs remain poorly understood. To address the molecular basis of this decline, we first collected skin from the footpads of ‘Tabby’ male mice, in which SWGs do not develop due to a spontaneous point mutation in the sex-linked gene *Eda* (*Eda*^-/Y^) [12], and from wild-type (WT) mice, where SWGs develop normally. Using RNA-sequencing (RNA-seq) analysis, we compared the transcriptomes of Tabby and WT mice to identify SWG-enriched mRNAs, and more precisely, a core of mRNAs encoding proteins responsible for the secretory function of SWGs [13]. We then examined the changes in the abundance of these mRNAs in young relative to old male mice and identified aging-related expression changes in 28 SWG-enriched mRNAs, including 11 core secretory mRNAs. Further investigation in SWGs of the proteins encoded by these mRNAs using immunofluorescence revealed that FOXC1 levels significantly increased in SWGs from older mice.

## RESULTS

### Sweating capacity is reduced in old mice

**Figure 1:**
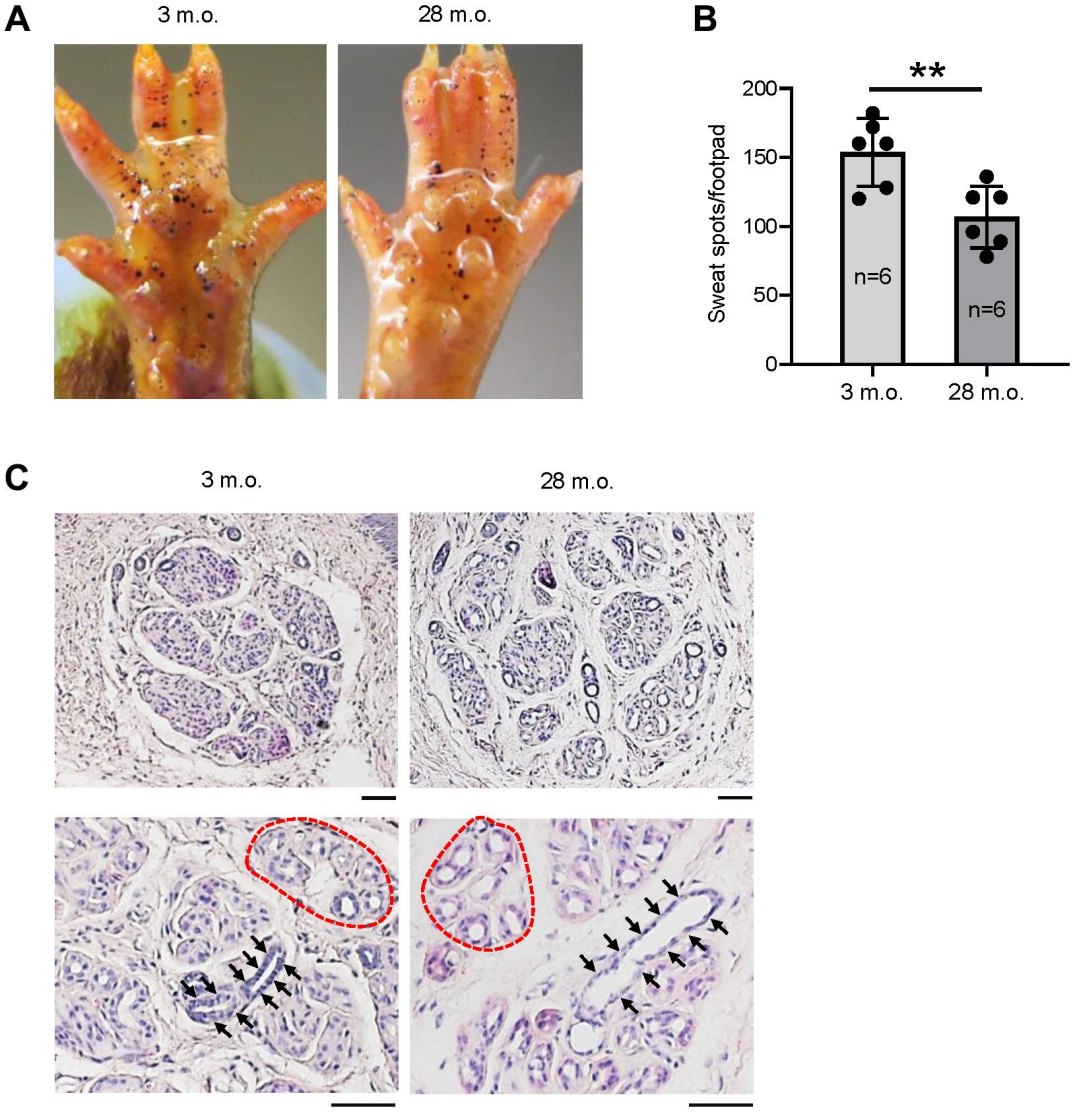
**The numbers of active SWGs decline in old relative to young mice.** (**A**) Iodine-starch sweat test to evaluate the number of sweating spots (black dots) in young (3 m.o.) and old (28 m.o.) male mice. (**B**) Quantitation of the number of sweating spots in sweat tests from panel (**A**). Quantification from 6 biological replicates each for young and old mice. (**C**) Distribution of SWGs in young and old mice (upper panels); old SWGs display enlarged ductal lumens (arrows) and secretory lumens (dotted circles, lower panels). Scale bars, 50 μm. Data in (**B**) represent the means and S.D. from six biological replicates; significance (^**^p < 0.01; ^***^p < 0.001) was established using Student’s *t*-test. Other data are representative of three or more biological replicates.

To evaluate the change of sweating capacity during aging, we studied 6 young (3 months old (m.o.)) and 6 old (28 m.o.) C57BL/6JN male mice using the iodine-starch sweat test [4]. The results revealed a 30% reduction of sweating spots in old relative to young mice (Figure 1A, 1B), indicative of reduced numbers of actively secreting SWGs in the old. The reduction of active SWG numbers is consistent with earlier reports in old mice and humans [7, 8, 14]. Histological analysis showed loosely distributed SWG clusters in old relative to young mice, but without significant reductions in cluster numbers (Figure 1C, upper panels). Notably, the lumens of sweat ducts (Figure 1C, arrows) and secretory coils (Figure 1C, dotted red circles as examples) were enlarged in old SWGs.

### Identification of mRNAs enriched in SWGs

The sweating phenotype in old mice prompted us to analyze the transcriptomic alterations accompanying SWG aging. To do so, we first identified mRNAs enriched in SWGs by studying male ‘Tabby’ mice, in which all parts of the SWG are absent, including the secretory coil as well as dermal and epidermal ducts, but the skin epidermis and dermis remain intact [13]. Thus, we performed RNA-seq analysis of skin obtained from the footpads (where the mouse SWGs are located) of SWG-deficient Tabby mice and SWG-bearing WT mice in order to identify mRNAs preferentially expressed in SWGs (GSE249784).

**Figure 2:**
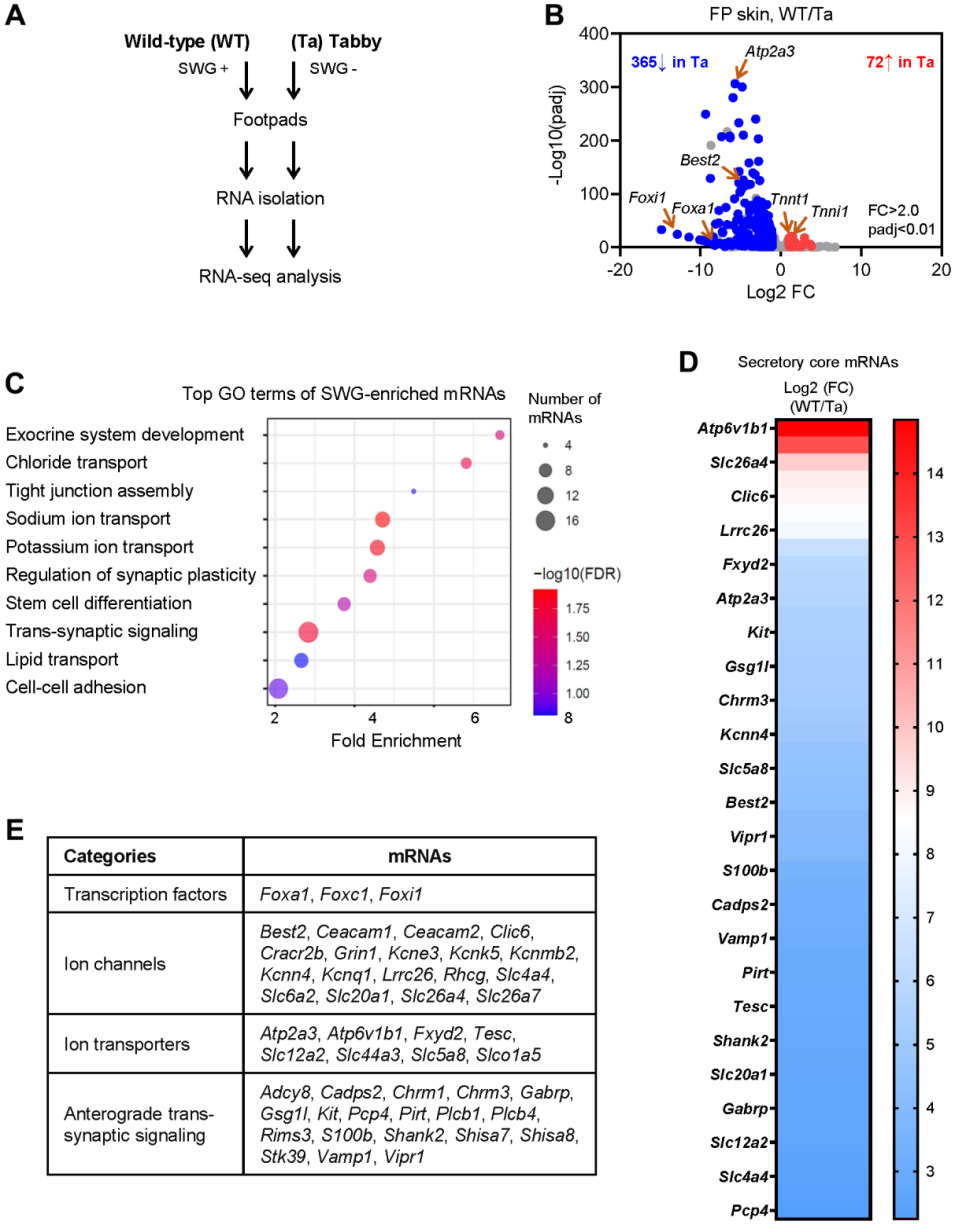
**Identification of SWG-enriched mRNAs and core secretory mRNAs in mice by RNA-seq analysis.** (**A**) Schematic of the RNA-seq analysis (GSE249784). (**B**) Volcano plot analysis of the differentially abundant mRNAs in WT and Tabby male footpad skin. (**C**) GO annotation of the top functional terms of SWG-enriched mRNAs. (**D**) Core secretory mRNAs and expression fold difference in footpad skin (Tabby relative to WT). (**E**) Functional categories of core secretory mRNAs.

Unlike the hind footpads, the fore footpads in mice lack hair follicles in the center [15]; thus, to avoid contamination of the hair follicle transcriptomes, we collected fore footpads from 3 m.o. Tabby male mice and age- and sex-matched WT mice (3 biological replicates each) for RNA-seq analysis (Figure 2A). At the established criteria of absolute fold change >2.0 and *p*-value < 0.01, 365 mRNAs were less abundant and 72 mRNAs more abundant in Tabby relative to WT footpads (Figure 2B and Supplementary Table 1). We focused on the 365 mRNAs showing reduced expression in the SWG-deficient Tabby footpads; we reasoned they would be enriched in mRNAs expressed in SWGs.

We narrowed down the list of underrepresented mRNAs by establishing a higher fold difference between Tabby and WT. The *Eda* mRNA in Tabby male footpads was ~4.5-fold lower than in WT footpads (Supplementary Table 1), and was virtually undetectable by *in situ* hybridization experiments [12]. Applying this criterion (Tabby/WT <4.5) yielded 171 underrepresented mRNAs in Tabby footpads (Supplementary Table 1); we designated these 171 mRNAs as ‘SWG-enriched’. As expected, GO annotation (using ShinyGO) showed that many of the SWG-enriched mRNAs were associated with exocrine system development, ion transport, and synaptic regulation, along with additional terms including tight junction assembly, stem cell differentiation, lipid transport, and cell-cell adhesion (Figure 2C).

### Identification of core secretory mRNAs

From the 171 mRNAs, we further selected mRNAs encoding proteins associated with secretion based on GO annotations (Figure 2C). In particular, 47 mRNAs were identified encoding proteins implicated in secretory functions, from transcription factors to ion channels, ion transporters, and trans-synaptic signaling proteins (Figure 2D, 2E and Supplementary Table 1); we named them ‘core secretory’ mRNAs. Many such mRNAs expressed in SWGs were present in this core list – *Foxa1*, *Best2*, *Foxc1*, *Foxi1*, *Kcnn4*, *Kcnk5*, *Kcnq1*, *Kcne3*, *Clic6*, *Slc26a4* (*Pendrin*), *Slc12a2* (*Nkcc1*), and *Chrm3* mRNAs were reported to be expressed specifically in SWGs in rodent skin [4, 15–18]. Interestingly, mice with mutations in genes *Foxa1*, *Best2*, and *Foxc1*, and mice with double mutations in genes *Kcnn4* and *Kcnk5*, were reported to have reduced sweat secretion [4, 16, 19]. However, most core secretory mRNAs, including *Atp6v1b1*, *Serca3*, *Kit*, *Fxyd2*, *Vipr1*, *Cadps2*, *Gsg1l*, and *Vamp1* mRNAs, have not been reported in SWGs (Figure 2D, 2E and Supplementary Table 1).

**Figure 3:**
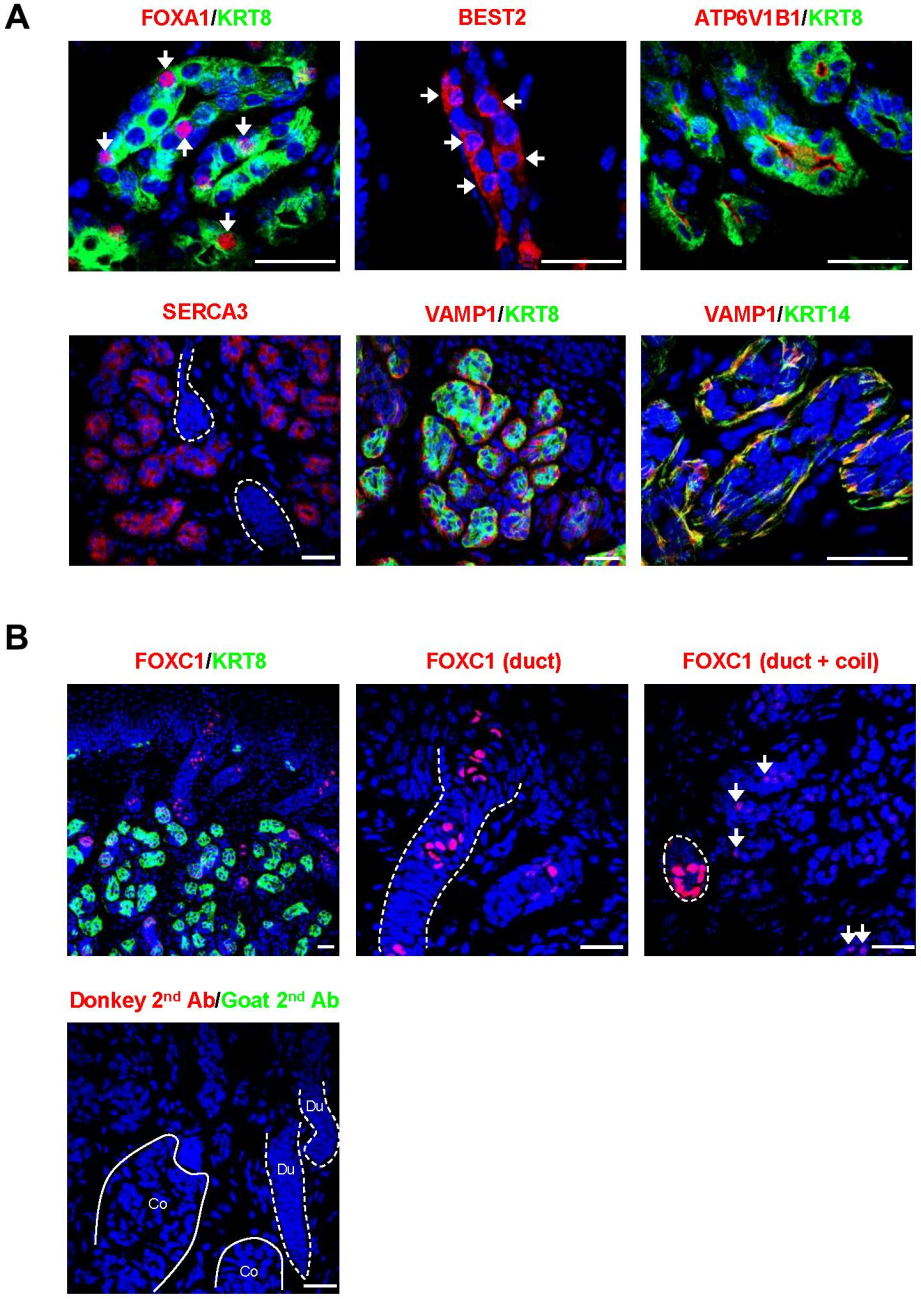
**Localization of core secretory proteins in SWGs.** (**A**) Localization of FOXA1 and BEST2 in scattered secretory cells (arrows), ATP6V1B1 in luminal cell membrane in the secretory coil, SERCA3 in the cytoplasm of secretory cells (but not sweat duct, demarcated by broken lines), and VAMP1 protein surrounding KRT8+ secretory cells and co-localizing with the myoepithelial cell marker KRT14. (**B**) *Top*, expression of FOXC1 in sweat duct cells (left); higher magnification shows localization of FOXC1 in epidermal and dermal duct luminal cells (middle). FOXC1 protein is expressed in secretory cells (arrows, right), but expression signals are stronger in duct cells (dotted circle). *Bottom*, negative control staining (secondary antibodies). Scale bars, 25 μm. Data are representative of two to four biological replicates.

We then confirmed the SWG-specific localization of core secretory proteins by immunofluorescence staining (Figure 3). The transcription factor FOXA1 and the anion channel BEST2 were expressed in scattered sweat secretory cells (Figure 3A), as previously reported [4]. Some proteins were also identified that were novel to SWGs (Figure 3A). For example, ATP6V1B1, a proton pump, was specifically expressed on the luminal membrane of secretory cells; ATP2A3 (SERCA3) was found in the cytoplasm of the secretory coil, but not the sweat duct; while VAMP1, a synaptic vesicle-associated protein, was localized in KRT14+ myoepithelial cells surrounding the secretory coil, suggesting that myoepithelial cells may be an important target of neuronal signaling in SWGs.

FOXC1 was strongly expressed in nuclei of luminal cells in epidermal and dermal sweat ducts, which is consistent with its critical function in sweat duct differentiation (Figure 3B) [19]. Notably, FOXC1 protein was also observed in the nuclei of scattered secretory cells, although the expression levels were much lower in the secretory coil compared to the duct (Figure 3B). The above core secretory proteins analyzed by fluorescence immunohistology were specifically localized in the secretory coil and/or sweat duct, but not in skin epidermis or dermis (not shown).

### Identification of altered SWG-enriched mRNAs in old mice

**Figure 4:**
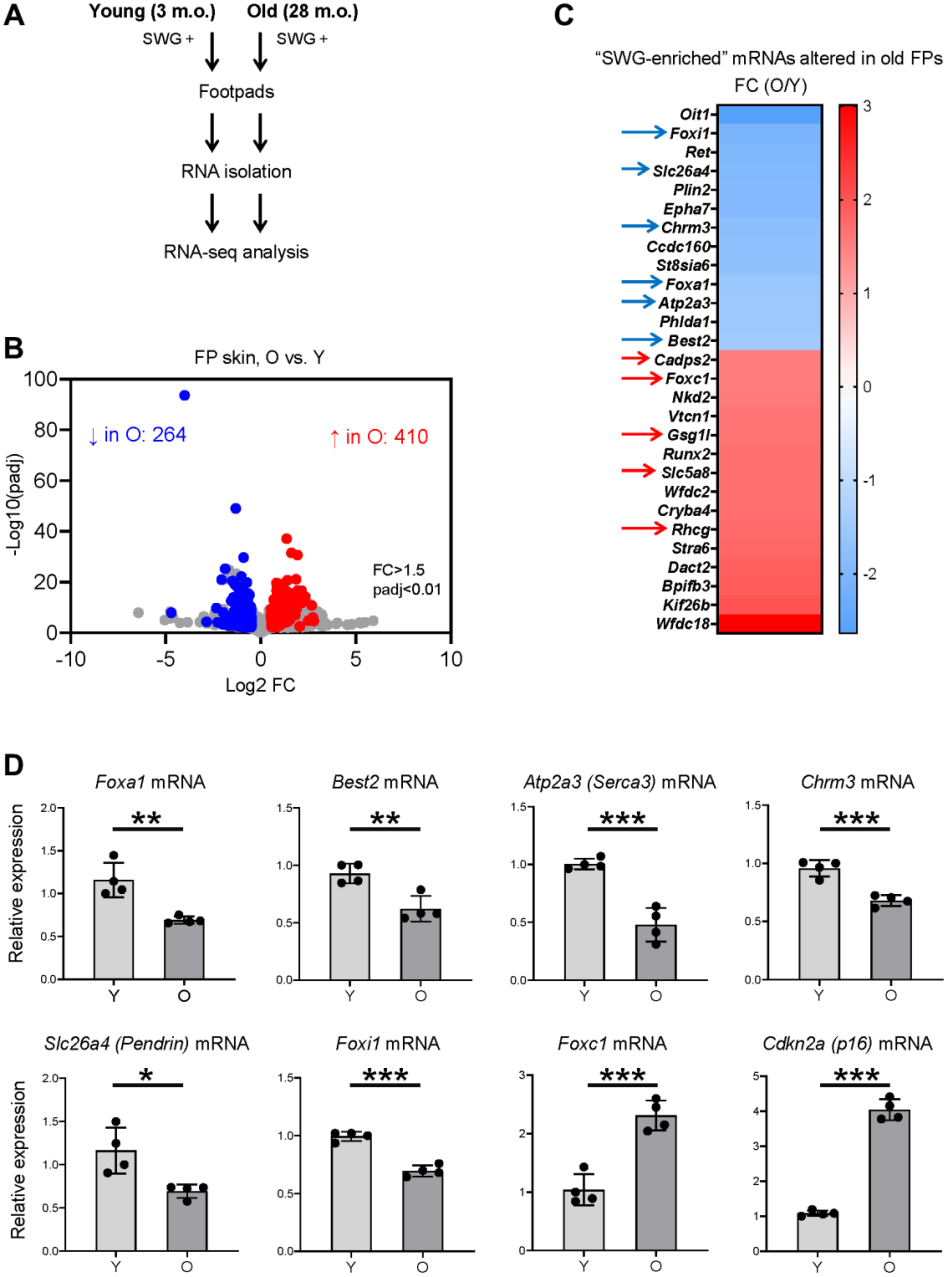
**Identification of SWG mRNAs displaying altered expression in old footpad skin.** (**A**) Schematic of the RNA-seq analysis. (**B**) Volcano plot of differentially expressed mRNAs in young (3 m.o.) versus old (28 m.o.) male footpad skin. (**C**) 28 SWG-enriched mRNAs showing differential abundance in old footpad skin; 11 core secretory mRNAs showing differential abundance in expression in old footpads (arrows). (**D**) RT-qPCR analysis of the expression levels of core secretory mRNAs and a senescent marker, *p16* mRNA, in young (3 m.o.) and old (28 m.o.) male footpad skin. Data in (**D**) represent the means and S.D. from four biological replicates; significance (^*^p < 0.05; ^**^p < 0.01; ^***^p < 0.001) was established using Student’s *t*-test.

We then determined if the SWG-enriched, core secretory mRNAs changed with age. We used RNA-seq analysis to identify differentially expressed mRNAs in the footpads of young (3 m.o.) and naturally aged (28 m.o.) male C57BL/6JN mice (4 biological replicates per group; Figure 4A). We found lower levels of 264 mRNAs and higher levels of 410 mRNAs in old relative to young footpads, using absolute fold change >1.5 and *p* < 0.01 (Figure 4B and Supplementary Table 2). The list included 28 SWG-enriched mRNAs, 13 less abundant and 15 more abundant in old footpads compared to young (Figure 4C, Supplementary Table 2), many of them core secretory mRNAs (see below). Among the non-secretory, SWG-enriched mRNAs reduced with age, *Oit1* mRNA encodes OIT1, a protein that promotes the integrity of the gastrointestinal tract [20], and *Plin2* mRNA encodes PLIN2, a protein implicated in lipid droplet formation [21]. Among the mRNAs increasing with age, *Stra6* mRNA encodes STRA6, a protein important for removing retinol from the retinol-retinol binding protein complex [22], and WFDC family proteins were proposed to be protease inhibitors [23]. The functions of these mRNAs in SWGs warrant further study.

### Identification of altered core secretory mRNAs in old mice

Notably, 11 of the 28 altered SWG-enriched mRNAs in old mice encoded core secretory proteins. *Foxa1*, *Best2*, *Chrm3*, *Atp2a3*, *Foxi1*, and *Slc26a4* mRNAs were less abundant, whereas *Foxc1*, *Gsg1l*, *Cadps2*, *Rhcg*, and *Slc5a8* mRNAs were more abundant in old compared to young footpads (Figure 4C, arrows, and Supplementary Table 2). These mRNAs encode transcription factors (FOXA1, FOXI1, FOXC1), ion channels (BEST2, SLC26A4, RHCG), ion transporters (ATP2A3/SERCA3, SLC5A8), and trans-synaptic signaling proteins (CHRM3, GSG1L, CADPS2), most of them with important roles in SWGs or other secretory organs (Figure 2E and Discussion).

The RNA-seq data were validated using reverse transcription (RT) followed by quantitative real-time PCR (RT-qPCR) analysis using specific primer pairs (Supplementary Table 3). As expected, there were moderate but significant reductions in the levels of *Foxa1*, *Best2*, *Atp2a3, Chrm3*, *Slc26a4*, and *Foxi1* mRNAs in old footpads (Figure 4D), while the levels of *Foxc1* mRNA were significantly elevated in old footpads (Figure 4D). As a marker of tissue aging, the senescent marker *p16* mRNA was elevated in old footpads by both RT-qPCR and RNA-seq analyses (Figure 4D and Supplementary Table 2).

**Figure 5:**
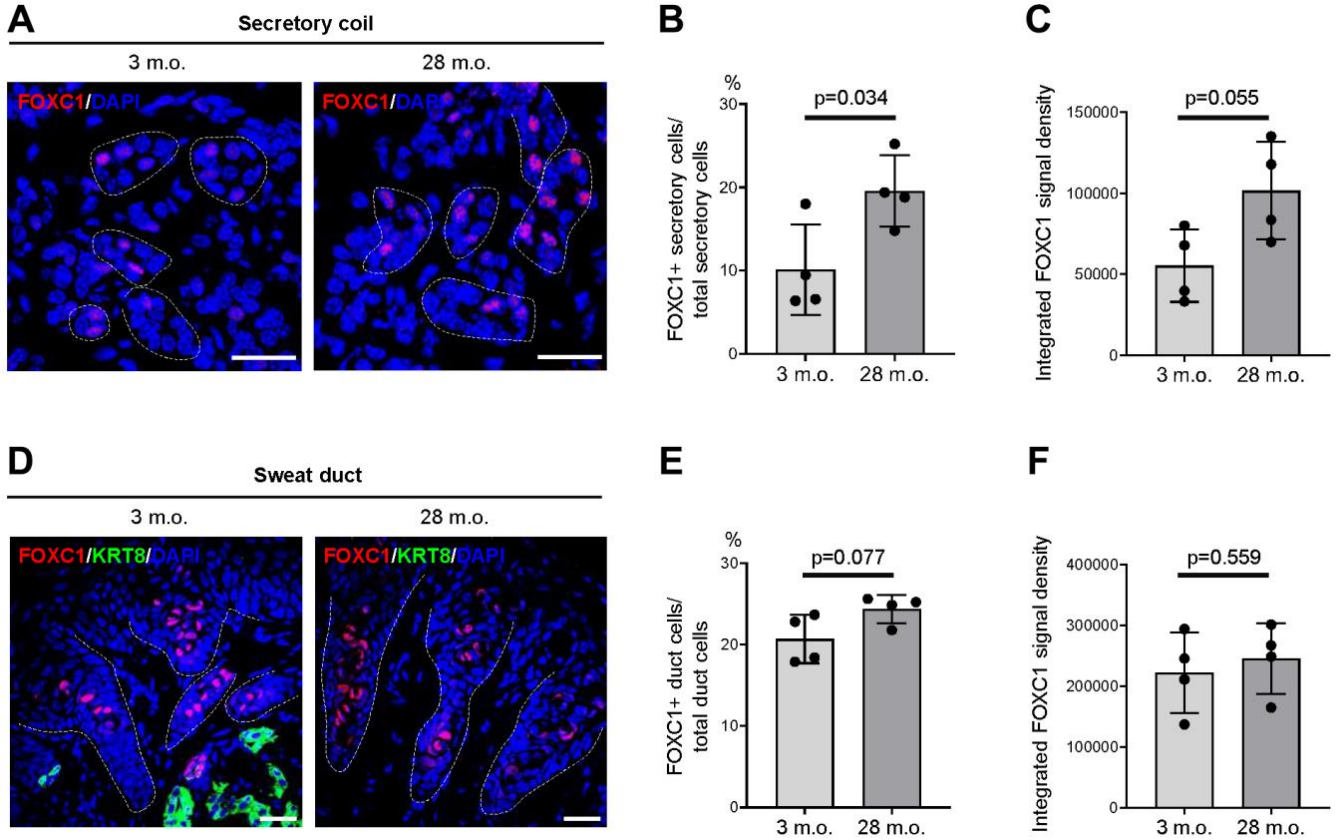
**Expression changes of FOXC1 protein in old SWGs.** (**A**–**C**) FOXC1+ secretory cells in young (3 m.o.) and old (28 m.o.) male SWGs were detected by immunofluorescence microscopy (**A**), and the numbers of FOXC1+ secretory cells (**B**), and average FOXC1 signal intensities (**C**) were calculated. (**D**–**F**) FOXC1 in the luminal cells of sweat ducts from young (3 m.o.) and old (28 m.o.) male SWGs were detected by immunofluorescence microscopy (**D**), and the numbers of FOXC1+ duct cells (**E**) and average FOXC1 signal intensities (**F**) were calculated. Scale bars, 25 μm. Data in (**B**, **C**, **E**, **F**) represent the means and S.D. from four biological replicates each for young and old; significance (^**^p < 0.01; ^***^p < 0.001) was established using Student’s *t*-test. Other data are representative of three or more biological replicates.

We then assessed whether the alterations in mRNA expression in old footpad skin resulted in detectable changes at the protein level using immunofluorescence staining and quantification of signal intensities. Most proteins, including FOXA1, BEST2, and SERCA3, did not show detectable changes in signal intensity or expression patterns between young and old SWGs (data not shown). However, a significant increase was observed in the number of FOXC1+ secretory cells in old SWGs (Figure 5A, 5B). The intensity of FOXC1 signals in secretory cells was also elevated overall in the old, although this parameter did not reach statistical significance (Figure 5C). Notably, FOXC1+ cell numbers and FOXC1 signal intensities were indistinguishable between young and old sweat ducts (Figure 5D–5F).

## DISCUSSION

The secretory function of SWGs declines with age [7, 8], but the underlying molecular mechanisms are poorly understood. In this study, we first obtained evidence that, in mouse, aging primarily reduced the number of active SWGs. We further identified mRNAs that were enriched in SWGs, including mRNAs specifically encoding secretory proteins whose expression levels changed with age. The ‘core secretory’ mRNAs altered in old SWGs encode both known and novel SWG proteins that may contribute to the age-related functional decline of SWGs, as we discuss below.

Among the mRNAs showing reduced abundance in old SWGs, *Foxa1*, *Best2*, and *Chrm3* mRNAs encode proteins that play key roles in sweat secretion. The FOXA1-BEST2 cascade is a master regulator of sweat secretion in mice [4], and their reduction in expression in old SWGs may directly contribute to the decrease of active SWGs (Figure 4C, 4D). CHRM3, but not CHRM1, mediates neuronal signaling to secretory activity in rat SWGs [17, 18], and its reduced abundance in old SWGs is likely implicated in the reduced sweat secretion of old mice. The functions of proteins encoded by other core secretory mRNAs that are less abundant with age, including SERCA3 (ATP2A3), SLC26A4 (Pendrin), and FOXI1, in SWGs remain unknown, although SERCA3 is a calcium pump involved in calcium influx from the cytoplasm into the endoplasmic reticulum (ER) [24]. Interestingly, the calcium sensor protein STIM1 regulates calcium influx from the interstitium to the ER [25], and thus SERCA3 may cooperate with STIM1 in storing calcium in the ER. Notably, intracellular calcium release from the ER upon CHRM3 activation is required for the initiation of sweat secretion [1], and IP3R2 was shown to mediate this release [26]. SERCA3, along with STIM1 and IP3R2, may regulate sweat secretion by controlling calcium flux, and its reduction in expression in old SWGs may affect secretory activities. SLC26A4 (Pendrin) is an anion channel involved in transporting chloride, bicarbonate, and iodide in the kidney and the inner ear, and its absence is responsible for hearing loss [27, 28], but its possible involvement in sweat secretion requires further study.

Among the mRNAs encoding secretory factors that showed higher abundance in old SWGs, the translation product of *Foxc1* mRNA, FOXC1, was found to regulate sweat duct differentiation, and its absence resulted in a sweat rash in mice [19]. We confirmed that FOXC1 protein is highly expressed in luminal cells of both epidermal and dermal ducts (Figure 3B), consistent with our earlier findings [19]. Surprisingly, we found that FOXC1 protein was also expressed in the nuclei of scattered secretory cells, although in much lower levels than in the luminal duct cells (Figure 3B). We further found that a significantly higher number of secretory cells express FOXC1 protein in old SWGs (Figure 5), although elucidating the function of FOXC1 in the secretory coil awaits further study. Some transcripts showing increased abundance in old SWGs, including *Gsg1l, Rhcg, Cadps2*, and *Slc5a8* mRNAs, were not previously identified in SWGs. *Gsg1l* mRNA encodes a suppressor of the glutamate receptor protein AMPAR, which mediates synaptic transmission in the brain [29]; high levels of GSG1L might similarly inhibit synaptic signals in old SWGs. RHCG, encoded by *Rhcg* mRNA, is a channel responsible for ammonia secretion in the collecting duct of the kidney [30, 31]. As sweat contains ammonia, which provides odor [32], elevated RHCG may increase odor in old mice. In sum, altered expression of novel mRNAs or proteins in old SWGs may contribute to the reduction of sweat secretion or altered sweat composition.

It is worth noting that the number of SWG-enriched, core secretory mRNAs in old SWGs was small, and their changes in abundance were also moderate. Similarly, we could not detect significant alterations in most of the encoded proteins in old SWGs. We believe the modest changes in mRNA and protein level reflect the moderate reduction (approximately 30%) of active SWGs in old mice. The altered expression levels of mRNAs and proteins in old SWGs may result from changes in cell phenotypes; for example, senescent cells, which display different gene expression programs, may accumulate with age in SWGs as indicated by the *p16* mRNA elevation in old SWGs (Figure 4D). Alternatively, they may reflect local changes in transcriptional programs (e.g., epigenetic modification of histones or DNA, altered transcription factor activity, etc.) or post-transcriptional programs directly affecting mRNAs (e.g., altered mRNA stability, localization, or translation) or modulating protein fate (e.g., altered protein stability, modification, processing, secretion, etc.) [33]. The molecular mechanisms that govern the expression levels of aging-associated mRNAs and proteins in SWGs also await further study.

In closing, we have investigated the phenotypic changes in SWGs in old mice, and have identified alterations in the expression levels of several SWG-enriched and core secretory mRNAs and proteins in this paradigm. Our study sets the stage for mechanistic studies of human SWGs, including investigating the levels of key mRNAs, the abundance of the encoded proteins, and the roles of these proteins in the impaired thermoregulation of older persons.

## MATERIALS AND METHODS

### Animal models

All mouse work was performed under an Animal Study Proposal (ASP#476-LGG-2024) approved by the Animal Care and Use Committee of the National Institute on Aging (NIA). C57BL/6JN male mice were imported from the NIA Aged Rodent Colonies (https://www.nia.nih.gov/research/dab/aged-rodent-colonies-handbook). *Eda* mutant Tabby mice (C57BL/6J *A^w-J^-Eda^Ta-6J^*/J, Strain#000338) were purchased from the Jackson Laboratory (Bar Harbor, ME), and bred in the NIA animal facility. All mice were provided with standard diet *ad libitum* and maintained under a 12-h/12-h light/dark cycle.

### Starch-iodine sweat test

The protocol of iodine-starch sweat test was reported previously [4]. Briefly, iodine/alcohol solution (1 g iodine/50 ml ethanol) was applied to the surface of a hind footpad of experimental mice. Once dry, the surface was painted with starch-oil (10 g starch/10 ml castor oil). Purple sweating spots start to form in 1–2 min and peak at around 10 min. Images were taken at 10 min with a close-up mode of a Canon PowerShot G16 camera and quantified using the ImageJ plug-in cell counter.

### Tissue collection from mice

Fore footpad skin was taken from 3 m.o. Tabby male mutant mice, as explained above (C57BL/6J *A^w-J^-Eda^Ta-6J^*/J, Strain#000338) and their wild-type (WT) littermates, as well as 3 m.o. and 28 m.o. (C57BL/6JN) male mice for histology and RNA isolation. For histological studies, tissues were embedded in a Tissue-Tek OCT compound (VWR, Cat#25608-930) on dry ice and stored in −80°C freezer or fixed in 10% formaldehyde (Ricca Chemical) for 16 h at 25°C, dehydrated, and embedded in paraffin. For RNA isolation, footpad skin was frozen on ice immediately after isolation and kept at −80°C until use.

### RNA isolation, library preparation, mRNA sequencing, and RT-qPCR analysis

Total RNA was extracted from frozen footpad skin obtained from three 3 m.o. Tabby male mice and three 3 m.o. WT male mice using Qiagen RNeasy Fibrous Tissue Mini Kit (Qiagen, Cat#74704) with DNase treatment. Briefly, tissue samples were transferred to 2.0 ml Sarstedt tubes (Sarstedt, Cat#72.694.006) containing 800 μl of RLT (RNA lysis buffer) (Qiagen, Cat#74704) plus DTT (Dithiothreitol) (Teknova, Cat#D9750) buffer from the Qiagen kit, and added 1.0-mm diameter Zirconia beads (Biospec, Cat#11079110z) to make a total volume of 1.25 ml. The tube was processed using the Precellys 24 homogenizer, followed by centrifugation at 17,000 RCF (g) for 5 min at 4°C. The resulting homogenate was evenly distributed into two 1.5-ml Eppendorf tubes for Proteinase K treatment, and subsequently used for RNA isolation. The quality and quantity of RNA were assessed using the Agilent Bioanalyzer and the RNA 6000 Nano Kit (Agilent, Cat#5067-1511).

An aliquot of RNA (125 ng) was utilized for sequencing library preparation using the Illumina TruSeq Stranded mRNA Library prep kit (Illumina, Cat# 20020594), following the manufacturer’s protocol. Briefly, mRNAs were isolated using Poly-T oligomer-containing magnetic beads, and subsequent cDNA synthesis was carried out. The cDNAs were subjected to 3’ end adenylation and adapter ligation, and were then purified using AMPure beads (Beckman, Cat#A63881). The purified cDNAs were size-selected using SPRIselect beads (Beckman, Cat#B23318), underwent PCR enrichment, and were purified once more with SPRIselect beads to generate the final libraries. The quality and quantity of the libraries were assessed using the Agilent DNA 1000 Screen Tape on the Agilent Tapestation. Finally, paired-end sequencing of the libraries was conducted using the Illumina NovaSeq 6000 sequencer. The same protocols were employed for isolating RNA and preparing cDNA libraries for four 3-m.o. and four 28-m.o. C57BL/6JN male mice. We obtained ~50 million reads from Tabby and WT footpad libraries, and ~40 million reads from young and old C57BL/6JN footpad libraries. RNA-seq data were deposited in GEO (GSE249784).

The RNA-seq reads were aligned to mouse genome NCBIM38/mm10 with spliced transcripts alignment to a reference (STAR) software v2.7.0f_0328, and subread (version 2.10.5) was used to create gene counts. Normalization of counts and differential expression analysis of genes between experimental groups were performed using the DESeq2 package (versions 1.30.1 and 1.36.0) pipeline [34].

For mRNA profiling of Tabby and WT controls, we set the criteria of absolute fold change (FC) >2.0, *p*-value < 0.01, and base mean >30, for significant change. From underrepresented mRNAs in Tabby footpads, we collected mRNAs that show FC >4.5 (WT/Ta) and designated them ‘SWG-enriched’ mRNAs. We carried out GO annotation (ShinyGO: http://bioinformatics.sdstate.edu/go/) [35] with SWG-enriched mRNAs, and further collected mRNAs encoding protein involved in secretory function. We designated them ‘core secretory’ mRNAs.

For mRNA profiling of young and old footpad skin, we set the criteria of absolute FC >1.5, *p*-value < 0.01, and base mean >50, as a significant change. We further identified SWG-enriched mRNAs and core secretory mRNAs from the significantly altered mRNA list in old footpad skin by Venn diagrams (https://bioinformatics.psb.ugent.be/webtools/Venn/).

Following RNA isolation from young and old mouse footpad skin, 500 ng of total RNA was used for reverse transcription (RT) followed by real-time quantitative PCR (qPCR) analysis. For qPCR analysis, 0.1 μl of cDNA was used with 250 nM of primers (Supplementary Table 3) and KAPA SYBR^®^ FAST qPCR Kits (KAPA Biosystems). RT-qPCR analysis was carried out on a QuantStudio 5 Real-Time PCR System (Thermo Fisher Scientific) with a cycle setup of 20 s at 95°C, followed by 40 cycles of 1 s at 95°C, and 20 s at 60°C. For the analysis, relative RNA levels were calculated after normalizing to mouse *Actb* mRNA (which encodes the housekeeping protein β-actin), using the 2^−ΔΔCt^ method.

### Histology, immunohistology, and quantification of signal intensities

For general histology, 5- to 8-μm paraffin sections were cut, deparaffinized, and stained with hematoxylin (Sigma-Aldrich, Cat#SLCL4498) and eosin (Sigma-Aldrich, Cat#SLCN7193) using the manufacturer’s standard protocol.

For immunofluorescence microscopy, 14-μm frozen sections were cut with a Leica CM3050S cryostat, attached to Silane-Prep Slides (Sigma-Aldrich, Cat#S4651-72EA), air-dried for 30 min, and stored at -20°C until use. Sections were fixed in 4% paraformaldehyde (PFA) (EMS, Cat#15710) for 6 min, rinsed in phosphate-buffered saline (PBS) (Sigma-Aldrich, Cat#P3813) containing 0.1% Triton X-100 (Sigma Aldrich, Cat#9036-19-5) for 15 min, and blocked with 10% donkey serum (DS) (Sigma-Aldrich, Cat#D9663-10 mL) in PBS + 0.1% Tween-20 (DS-PBST) (Sigma-Aldrich, Cat#P1379) for 1 h. Sections were incubated for 18 h at 4°C with primary antibodies diluted in DS-PBST (see Supplementary Table 3 for antibodies and dilutions). Sections were then rinsed with PBST and incubated with Alexa Fluor-conjugated secondary antibodies diluted in DS-PBST (see Supplementary Table 3) for 45 min at 25°C. Sections were mounted in ProLong Gold with DAPI (Invitrogen, Cat#P36935) and images taken with a Zeiss 980 LMS confocal microscope with settings of the channels of interest held constant. The z-stack function was used to create a z-stack image with slices width determined by the manufacturer’s “optimal” setting slices.

Signal quantification was performed using the ImageJ (https://imagej.net/ij/index.html) software following the method described by Shihan et al. [36] with modifications. Briefly, positive signals in the form of integrated density were measured. Maximum intensity (MAX) projections were created for each section. MAX projections for FOXC1 duct and secretory coil images were produced from 45 and 30 0.3-μm slices, respectively. Regions of interest (ROIs) for measurement were then identified by isolating, blurring, and thresholding the channel of interest. Each image set was processed with a unique constant threshold and blur setting. After creating a thresholding image, particle analysis was used to identify areas of interest. Two to four images were analyzed from each mouse for each unique image set. For FOXC1 quantification, >70 positive nuclei for ducts and >90 positive nuclei for secretory coil cells from each mouse were counted and averaged to obtain a final representative integrated density measurement. All measurements were adjusted to account for background signal by measuring the integrated density value of 6 randomly placed ROIs in control sections stained solely with secondary antibodies. ROIs used were of the approximately same size to the average positive ROIs. Representative measurements were then averaged to obtain an overall group average. Cells were counted for percentile quantification from MAX projections with the ImageJ plug-in cell counter. Statistical significance (^*^p < 0.05; ^**^p < 0.01; ^***^p < 0.001) was established using Student’s *t*-test.

## Supplementary Materials

Supplementary Table 1

Supplementary Table 2

Supplementary Table 3

## References

[1] Cui CY, Schlessinger D. Eccrine sweat gland development and sweat secretion. Exp Dermatol. 2015; 24:644–50. 10.1111/exd.12773 26014472 PMC5508982

[2] Sato K, Kang WH, Saga K, Sato KT. Biology of sweat glands and their disorders. I. Normal sweat gland function. J Am Acad Dermatol. 1989; 20:537–63. 10.1016/s0190-9622(89)70063-3 2654204

[3] Baker LB. Physiology of sweat gland function: The roles of sweating and sweat composition in human health. Temperature (Austin). 2019; 6:211–59. 10.1080/23328940.2019.1632145 31608304 PMC6773238

[4] Cui CY, Childress V, Piao Y, Michel M, Johnson AA, Kunisada M, Ko MS, Kaestner KH, Marmorstein AD, Schlessinger D. Forkhead transcription factor FoxA1 regulates sweat secretion through Bestrophin 2 anion channel and Na-K-Cl cotransporter 1. Proc Natl Acad Sci U S A. 2012; 109:1199–203. 10.1073/pnas.1117213109 22223659 PMC3268268

[5] Li H, Chen L, Zhang M, Xie S, Cheng L. Expression and localization of Forkhead transcription factor A1 in the three-dimensional reconstructed eccrine sweat glands. Acta Histochem. 2018; 120:520–4. 10.1016/j.acthis.2018.06.003 29909922

[6] Zhao J, Zhang L, Du L, Chen Z, Tang Y, Chen L, Liu X, You L, Zhang Y, Fu X, Li H. Foxa1 mediates eccrine sweat gland development through transcriptional regulation of Na-K-ATPase expression. Braz J Med Biol Res. 2022; 55:e12149. 10.1590/1414-431X2022e12149 35976271 PMC9377534

[7] Coull NA, West AM, Hodder SG, Wheeler P, Havenith G. Body mapping of regional sweat distribution in young and older males. Eur J Appl Physiol. 2021; 121:109–25. 10.1007/s00421-020-04503-5 32990756 PMC7815578

[8] Inoue Y, Shibasaki M, Ueda H, Ishizashi H. Mechanisms underlying the age-related decrement in the human sweating response. Eur J Appl Physiol Occup Physiol. 1999; 79:121–6. 10.1007/s004210050485 10029332

[9] Vicedo-Cabrera AM, Scovronick N, Sera F, Royé D, Schneider R, Tobias A, Astrom C, Guo Y, Honda Y, Hondula DM, Abrutzky R, Tong S, de Sousa Zanotti Stagliorio Coelho M, et al. The burden of heat-related mortality attributable to recent human-induced climate change. Nat Clim Chang. 2021; 11:492–500. 10.1038/s41558-021-01058-x 34221128 PMC7611104

[10] Ballester J, Quijal-Zamorano M, Méndez Turrubiates RF, Pegenaute F, Herrmann FR, Robine JM, Basagaña X, Tonne C, Antó JM, Achebak H. Heat-related mortality in Europe during the summer of 2022. Nat Med. 2023; 29:1857–66. 10.1038/s41591-023-02419-z 37429922 PMC10353926

[11] Ezure T, Amano S, Matsuzaki K. Aging-related shift of eccrine sweat glands toward the skin surface due to tangling and rotation of the secretory ducts revealed by digital 3D skin reconstruction. Skin Res Technol. 2021; 27:569–75. 10.1111/srt.12985 33576542 PMC8359204

[12] Srivastava AK, Pispa J, Hartung AJ, Du Y, Ezer S, Jenks T, Shimada T, Pekkanen M, Mikkola ML, Ko MS, Thesleff I, Kere J, Schlessinger D. The Tabby phenotype is caused by mutation in a mouse homologue of the EDA gene that reveals novel mouse and human exons and encodes a protein (ectodysplasin-A) with collagenous domains. Proc Natl Acad Sci U S A. 1997; 94:13069–74. 10.1073/pnas.94.24.13069 9371801 PMC24264

[13] Cui CY, Kunisada M, Esibizione D, Douglass EG, Schlessinger D. Analysis of the temporal requirement for eda in hair and sweat gland development. J Invest Dermatol. 2009; 129:984–93. 10.1038/jid.2008.318 18923450 PMC5155335

[14] Vilches JJ, Ceballos D, Verdú E, Navarro X. Changes in mouse sudomotor function and sweat gland innervation with ageing. Auton Neurosci. 2002; 95:80–7. 10.1016/s1566-0702(01)00359-9 11871787

[15] Kunisada M, Cui CY, Piao Y, Ko MS, Schlessinger D. Requirement for Shh and Fox family genes at different stages in sweat gland development. Hum Mol Genet. 2009; 18:1769–78. 10.1093/hmg/ddp089 19270025 PMC2671986

[16] Cui CY, Sima J, Yin M, Michel M, Kunisada M, Schlessinger D. Identification of potassium and chloride channels in eccrine sweat glands. J Dermatol Sci. 2016; 81:129–31. 10.1016/j.jdermsci.2015.11.001 26627722 PMC5526211

[17] Grant MP, Landis SC. Developmental expression of muscarinic cholinergic receptors and coupling to phospholipase C in rat sweat glands are independent of innervation. J Neurosci. 1991; 11:3772–82. 10.1523/JNEUROSCI.11-12-03772.1991 1744689 PMC6575287

[18] Grant MP, Landis SC, Siegel RE. The molecular and pharmacological properties of muscarinic cholinergic receptors expressed by rat sweat glands are unaltered by denervation. J Neurosci. 1991; 11:3763–71. 10.1523/JNEUROSCI.11-12-03763.1991 1744688 PMC6575272

[19] Cui CY, Ishii R, Campbell DP, Michel M, Piao Y, Kume T, Schlessinger D. Foxc1 Ablated Mice Are Anhidrotic and Recapitulate Features of Human Miliaria Sweat Retention Disorder. J Invest Dermatol. 2017; 137:38–45. 10.1016/j.jid.2016.08.012 27592801 PMC5183533

[20] Liang W, Peng X, Li Q, Wang P, Lv P, Song Q, She S, Huang S, Chen K, Gong W, Yuan W, Thovarai V, Yoshimura T, et al. FAM3D is essential for colon homeostasis and host defense against inflammation associated carcinogenesis. Nat Commun. 2020; 11:5912. 10.1038/s41467-020-19691-z 33219235 PMC7679402

[21] Mishra A, Liu S, Promes J, Harata M, Sivitz W, Fink B, Bhardwaj G, O'Neill BT, Kang C, Sah R, Strack S, Stephens S, King T, et al. Perilipin 2 downregulation in β cells impairs insulin secretion under nutritional stress and damages mitochondria. JCI Insight. 2021; 6:144341. 10.1172/jci.insight.144341 33784258 PMC8262280

[22] Chen Y, Clarke OB, Kim J, Stowe S, Kim YK, Assur Z, Cavalier M, Godoy-Ruiz R, von Alpen DC, Manzini C, Blaner WS, Frank J, Quadro L, et al. Structure of the STRA6 receptor for retinol uptake. Science. 2016; 353:aad8266. 10.1126/science.aad8266 27563101 PMC5114850

[23] Kirchhoff C, Habben I, Ivell R, Krull N. A major human epididymis-specific cDNA encodes a protein with sequence homology to extracellular proteinase inhibitors. Biol Reprod. 1991; 45:350–7. 10.1095/biolreprod45.2.350 1686187

[24] Anger M, Samuel JL, Marotte F, Wuytack F, Rappaport L, Lompré AM. The sarco(endo)plasmic reticulum Ca(2+)-ATPase mRNA isoform, SERCA 3, is expressed in endothelial and epithelial cells in various organs. FEBS Lett. 1993; 334:45–8. 10.1016/0014-5793(93)81677-r 8224225

[25] Cui CY, Noh JH, Michel M, Gorospe M, Schlessinger D. STIM1, but not STIM2, Is the Calcium Sensor Critical for Sweat Secretion. J Invest Dermatol. 2018; 138:704–7. 10.1016/j.jid.2017.09.038 29054597 PMC5828914

[26] Klar J, Hisatsune C, Baig SM, Tariq M, Johansson AC, Rasool M, Malik NA, Ameur A, Sugiura K, Feuk L, Mikoshiba K, Dahl N. Abolished InsP3R2 function inhibits sweat secretion in both humans and mice. J Clin Invest. 2014; 124:4773–80. 10.1172/JCI70720 25329695 PMC4347256

[27] Albert S, Blons H, Jonard L, Feldmann D, Chauvin P, Loundon N, Sergent-Allaoui A, Houang M, Joannard A, Schmerber S, Delobel B, Leman J, Journel H, et al. SLC26A4 gene is frequently involved in nonsyndromic hearing impairment with enlarged vestibular aqueduct in Caucasian populations. Eur J Hum Genet. 2006; 14:773–9. 10.1038/sj.ejhg.5201611 16570074

[28] Soleimani M. The multiple roles of pendrin in the kidney. Nephrol Dial Transplant. 2015; 30:1257–66. 10.1093/ndt/gfu307 25281699 PMC4513892

[29] Gu X, Mao X, Lussier MP, Hutchison MA, Zhou L, Hamra FK, Roche KW, Lu W. GSG1L suppresses AMPA receptor-mediated synaptic transmission and uniquely modulates AMPA receptor kinetics in hippocampal neurons. Nat Commun. 2016; 7:10873. 10.1038/ncomms10873 26932439 PMC4778064

[30] Biver S, Belge H, Bourgeois S, Van Vooren P, Nowik M, Scohy S, Houillier P, Szpirer J, Szpirer C, Wagner CA, Devuyst O, Marini AM. A role for Rhesus factor Rhcg in renal ammonium excretion and male fertility. Nature. 2008; 456:339–43. 10.1038/nature07518 19020613

[31] Lee HW, Verlander JW, Bishop JM, Igarashi P, Handlogten ME, Weiner ID. Collecting duct-specific Rh C glycoprotein deletion alters basal and acidosis-stimulated renal ammonia excretion. Am J Physiol Renal Physiol. 2009; 296:F1364–75. 10.1152/ajprenal.90667.2008 19321595 PMC2692449

[32] Czarnowski D, Górski J, Jóźwiuk J, Boroń-Kaczmarska A. Plasma ammonia is the principal source of ammonia in sweat. Eur J Appl Physiol Occup Physiol. 1992; 65:135–7. 10.1007/BF00705070 1396636

[33] López-Otín C, Blasco MA, Partridge L, Serrano M, Kroemer G. Hallmarks of aging: An expanding universe. Cell. 2023; 186:243–78. 10.1016/j.cell.2022.11.001 36599349

[34] Love MI, Huber W, Anders S. Moderated estimation of fold change and dispersion for RNA-seq data with DESeq2. Genome Biol. 2014; 15:550. 10.1186/s13059-014-0550-8 25516281 PMC4302049

[35] Ge SX, Jung D, Yao R. ShinyGO: a graphical gene-set enrichment tool for animals and plants. Bioinformatics. 2020; 36:2628–9. 10.1093/bioinformatics/btz931 31882993 PMC7178415

[36] Shihan MH, Novo SG, Le Marchand SJ, Wang Y, Duncan MK. A simple method for quantitating confocal fluorescent images. Biochem Biophys Rep. 2021; 25:100916. 10.1016/j.bbrep.2021.100916 33553685 PMC7856428

