# Supplementary material for: Characterization of age-associated gene expression changes in mouse sweat glands
Source: Aging (Albany NY). 2024 Apr 17;16(8):6717–30. doi: 10.18632/aging.205776 (PMC11087089; doi:10.18632/aging.205776)
Supplement: Supplementary Table 3 [file aging-16-205776-s003.pdf]

SUPPLEMENTARY TABLE

Supplementary Table 3. Primers used for RT-qPCR analysis.

Genes	Primer sequence
mActb_F	TTCTTTGCAGCTCCTTCGTT
mActb_R	ATGGAGGGGAATACAGCCC
mFoxa1_F	GAGGGTTTTGTCTGCATGGC
mFoxa1_R	ACTGGGGAAAATTGTGCGTG
mBest2_F	TCTCTGTCCCTTGTGTCCCTGT
mBest2_R	CACTCTGGCTGTGTAGGTGA
mAtp2a3_F	ATGACTGCAGCCGGTTTGTA
mAtp2a3_R	CTCTGGTCTCGGTGGGTCTA
mChrm3_F	GGGGAACTTAGCCTGTGACC
mChrm3_R	CGGCTCGTTTTGTTGTTCGT
mSlc26a4_F	CTGGTCGGGCAGAACTCCG
mSlc26a4_R	CGACGAGGGATGACGTTCG
mFoxi1_F	AACAATGGCTTGGAGGGAGG
mFoxi1_R	TCACAAAAGAGGAGCGAGCA
mFoxc1_F	TCATTCGTCTTGCCCCTCTG
mFoxc1_R	ACTTTCTGGTGTTTGGTCCGT
mCdkn2a_F	TGTTGAGGCTAGAGAGGATCTTG
mCdkn2a_R	CGAATCTGCACCGTAGTTGAGC
